# Risks predicting prolonged hospital discharge boarding in a regional acute care hospital

**DOI:** 10.1186/s12913-018-2879-2

**Published:** 2018-01-30

**Authors:** Sajid A. Shaikh, Richard D. Robinson, Radhika Cheeti, Shyamanand Rath, Chad D. Cowden, Frank Rosinia, Nestor R. Zenarosa, Hao Wang

**Affiliations:** 1 Department of Information Technology, John Peter Smith Health Network, 1500 S. Main St., Fort Worth, TX 76104 USA; 2 Department of Emergency Medicine, Integrative Emergency Services, John Peter Smith Health Network, 1500 S. Main St., Fort Worth, TX 76104 USA; 3 Department of Quality Office, John Peter Smith Health Network, 1500 S. Main St., Fort Worth, TX 76104 USA

**Keywords:** Hospital discharge, Boarding time, Disposition, Consultation

## Abstract

**Background:**

Prolonged hospital discharge boarding can impact patient flow resulting in upstream Emergency Department crowding. We aim to determine the risks predicting prolonged hospital discharge boarding and their direct and indirect effects on patient flow.

**Methods:**

Retrospective review of a single hospital discharge database was conducted. Variables including type of disposition, disposition boarding time, case management consultation, discharge medications prescriptions, severity of illness, and patient homeless status were analyzed in a multivariate logistic regression model. Hospital charges, potential savings of hospital bed hours, and whether detailed discharge instructions provided adequate explanations to patients were also analyzed.

**Results:**

A total of 11,527 admissions was entered into final analysis. The median discharge boarding time was approximately 2 h. Adjusted Odds Ratio (AOR) of patients transferring to other hospitals was 7.45 (95% CI 5.35–10.37), to court or law enforcement custody was 2.51 (95% CI 1.84–3.42), and to a skilled nursing facility was 2.48 (95% CI 2.10–2.93). AOR was 0.57 (95% CI 0.47–0.71) if the disposition order was placed during normal office hours (0800–1700). AOR of early case management consultation was 1.52 (95% CI 1.37–1.68) versus 1.73 (95% CI 1.03–2.89) for late consultation. Eighty-eight percent of patients experiencing discharge boarding times within 2 h of disposition expressed positive responses when questioned about the quality of explanations of discharge instructions and follow-up plans based on satisfaction surveys. Similar results (86% positive response) were noted among patients whose discharge boarding times were prolonged (> 2 h, *p* = 0.44). An average charge of $6/bed/h was noted in all hospital discharges. Maximizing early discharge boarding (≤ 2 h) would have resulted in 16,376 hospital bed hours saved thereby averting $98,256.00 in unnecessary dwell time charges in this study population alone.

**Conclusion:**

Type of disposition, case management timely consultation, and disposition to discharge dwell time affect boarding and patient flow in a tertiary acute care hospital. Efficiency of the discharge process did not affect patient satisfaction relative to the perceived quality of discharge instruction and follow-up plan explanations. Prolonged disposition to discharge intervals result in unnecessary hospital bed occupancy thereby negatively impacting hospital finances while delivering no direct benefit to patients.

## Background

Emergency Department (ED) crowding is now a global concern [1, 2]. Interventions to decrease ED crowding are routinely developed and implemented [3, 4]. Reducing and eliminating ED boarding time thereby minimizing the numbers of boarders is one of the targets to reduce relative crowding [5–7]. Strategies to reduce / eliminate ED boarding include moving boarders to inpatient unit hallways [6, 8] and transferring boarders to ED observation units or admission holding units [9–11]. These interventions are based on relative overall hospital resources and fail to provide ongoing functional capacity when overall capacity is breached. Any intervention and/or combination of interventions eventually approaches futility when ED outflow pathways are completely obstructed.

An important step in avoidance of ED crowding is high efficiency release of hospital beds driven by reduction of the disposition to discharge interval. Recent studies investigated the final bottleneck of ED outflow pathways by identifying inpatients experiencing delayed discharge from the hospital [5, 12]. Cross-sectional computer model analysis demonstrated the potential to reduce ED boarding by improving the hospital discharge process [13]. Similar modeling illustrated an anticipated 27–57% decrease in ED borders and a reduction of 7–14 h on an average ED length of stay in the setting of a high efficiency hospital discharge process [14]. Hospital metrics provide evidence that delayed inpatient discharges directly contribute to ED crowding [5]. Therefore, it is worthwhile to identify risks affecting delayed inpatient discharge.

In general, delayed discharge refers to prolonged length of stay occurring at any point along the patient care timeline whether during ED or inpatient care intervals [15, 16]. This study focuses on the last step of patient hospitalization (i.e., hospital discharge boarding). Hospital discharge boarding time is defined as the discharge disposition order to patient departure time interval. Given that the study hospital utilizes a discharge lounge where some patients may await transportation once released from their inpatient unit (i.e., bed) we modified the discharge boarding time definition to be the discharge disposition order to end of inpatient bed occupancy interval.

Significantly prolonged discharge boarding intervals impact availability of hospital beds thereby obstructing ED outflow of recently admitted patients. Unfortunately, such delays rarely studied in the literature. Several studies identified the risks of prolonged boarding when considering specific patient populations but still unable to show the direct link to prolonged hospital discharge boarding. Tucker et al. reported delayed hospital discharge intervals among psychiatric patients with cognitive impairment and/or requiring arrangement of social care [17]. Challis et al. categorized hospitalization into four sequential framework including preadmission, admission, mode of care in hospital, and discharge arrangement. Authors studied particularly on discharge arrangement framework and reported that such delays are common when arranging a care home or requiring a transition to home health care among geriatric patient populations [18]. Little is known beyond these psychosocial risks of other potential variables affecting prolonged boarding time in an adult general hospital.

The aim of this study is to (1) identify the status of discharge boarding time at the study hospital; (2) determine the independent risks affecting prolonged discharge boarding; and (3) estimate the potential outcomes related to delayed discharge intervals. Only through understanding the relative impact of individual contributors to delayed discharge intervals can meaningful offsetting interventions be developed, implemented, and tracked to arrive at a more resource efficient and fiduciarily responsible future state that better serves our patients.

## Methods

### Study design and patient population

This is a retrospective single center observational project. The study hospital is a publicly funded urban tertiary care referral center with a total of 537 licensed beds serving approximately 2.5 million residents and supporting various charitable programs. It is a regional Level 1 trauma center, chest pain center, and comprehensive stroke center. The study population have relatively high psychosocial risks without sufficient financial support. The local institutional review board reviewed the protocol and approved this study with a waiver of informed consent.

### Inclusion and exclusion criteria

Data of interest covering the period Jan 1, 2016 through Jun 30, 2016 was retrieved from the study hospital electronic medical record (EMR). All patients discharged directly from the study hospital were included in this study. We included all admissions for any given patients during the study period as these represent separate and distinct encounters for any single patient. Patients admitted to the hospital that were subsequently discharged from the Emergency Department (ED) having never physically transitioned to an inpatient unit were excluded. Our study specifically focused on discharge process workflows of the inpatient setting therefore those whose hospitalization did not include the inpatient discharge process were excluded. The ED discharge process is sufficiently different (i.e., more efficient) from the inpatient process that inclusion of the above cohort in final analysis would significantly impact results leading to inaccuracy regarding the study objectives of interest. Patients that expired during ED or Intensive Care Unit (ICU) were also excluded due to the relatively different procedures (e.g., infection control, decontamination, coroner or law enforcement involvement, etc.) as compared to other inpatient units. Additionally, patients whose discharge boarding times were indeterminate were also excluded from this study.

### Variables explanations

Patient basic demographic data including age, gender, race, and ethnicity were analyzed in this study. Discharge boarding time was defined as the discharge disposition order time to end of inpatient bed occupancy interval. Potential risks contributing to prolonged discharge boarding were discussed with hospital administration. The following variables were considered as significant pre-test contributors to prolonged discharge boarding time: (1) specific disposition (e.g., discharge to home, discharge to assisted living facility, etc.); (2) facilitation of immediate post-discharge follow-up (e.g., primary care physician appointment, specialist appointment, allied health appointment, etc.); (3) number of medications prescribed upon final disposition; (4) case manager and/or social worker consultation requirement during hospitalization; (5) time of disposition order placement in EMR; (6) homeless status upon discharge; and (7) severity of patient illness. Final dispositions included direct discharge to home without further assistance, discharge home with home health care service requirement, discharge home with hospice service requirement, transfer to skilled nursing facility, transfer to court or law enforcement custody, or expired while receiving care in a non-critical care hospital inpatient unit. If case mangers or social workers were consulted during hospitalization including, but not limited for, assisting for financial support, facilitating home health service, providing transportations, or arranging placement after the index hospital discharge, the time interval between the consultation order and the final disposition order was calculated. If multiple consult orders were placed, only the one timed closest to the final disposition order was chosen for analysis. Early consultation was defined as a consult order placed more than 24 h prior to final disposition. Late consultation was defined as a consult order placed less than 24 h prior to final disposition. Disposition order time was categorized based on EMR placement as having occurred within regular office hours (0800–1700) versus non-office hours (1701–0759).

Homeless patients at our local publicly funded county hospital network were identified in our EMR by using the keywords “homeless status” and pairing those positive queries with the Tarrant County Homeless Management Information System (HMIS) database that contains personal information of individuals meeting the US Department of Housing and Urban Development (HUD) definition of homelessness at the time of entry into the system. We issued a card to each person entered into the HMIS and entitled them access to homeless shelters and social services for 12 months. Individual HMIS information was matched with “homeless status” located in the EMR and verified using personal health information. When the data between the two datasets aligned, a flag was created and used to identify homeless patients contained within the overall study census.

Severity of illness (SOI) was categorized based on the All Patient Refined Diagnosis Related Group (APR-DRG) for each patient entered in the study. APR-DRG was developed by 3 M Health Information Systems in a joint effort with National Association of Children’s Hospitals and Related Institutions [19]. Its initial purpose was to properly determine the appropriate value of care for higher acuity patients thereby providing a better model for predicting resource needs [20]. APR-DRG is a clinical model and is disease specific. Each APR-DRG is subdivided into four severity of illness (SOI) categories including minor, moderate, major, and extreme. SOI is calculated based on patient age, primary diagnosis along with severity of secondary diagnoses. Therefore, SOI determines overall patient severity of illness according to the extent of physiological decomposition or organ system loss of function.

### Outcome measurements

Prolonged discharge boarding time was used as the primary outcome measurement. Variations in initial severity versus resolution of disease often requires patient transition across several care acuity environments (e.g., ICU, telemetry, medical / surgical unit). Discharge boarding time specifically refers to the discharge disposition order time to end of inpatient bed occupancy interval within the last segment of a hospitalization encounter regardless of the specific unit from which the final disposition occurred. Our secondary outcome analyzed whether quality of discharge and follow-up instruction explanation was impacted by relative discharge boarding time (i.e., normal versus prolonged). These responses were collected from patient satisfaction surveys (National Research Corporation) that specifically queried whether doctors, nurses or other hospital staff talked with the patient about post-discharge needs and assistance resources. We also measured specific charges during the last segment of hospitalization (i.e., start of last inpatient bed occupancy to end of last inpatient bed occupancy immediately following discharge disposition interval). Finally, we estimated potential hospital bed hours saved if all patients completed discharge boarding within 2 h of discharge disposition order placement.

### Study protocol

Expected discharge boarding time was discussed in depth with hospital administration. Median boarding time was reported simultaneously during the discussion. A modified Delphi survey reported that 2 h discharge boarding time (i.e., discharge disposition order time to end of inpatient bed occupancy interval) was considered reasonable cut-point marker, easy to report, and more pragmatic for future implementing interventions. Therefore, two groups (regular [≤ 2 h] versus prolonged [> 2 h] discharge boarding) of patients were entered into the final analysis. Variables including patient basic demographic data and those predetermined as significant pre-test contributors (see Variables Explanation section) to prolonged discharge boarding time were analyzed between these two groups. Independent risks affecting prolonged boarding were determined. Additionally, we compared the percentage of positive (i.e., “yes”) responses from patient after-care satisfaction surveys regarding quality of the explanations of discharge instructions and follow-up plans between the two groups. Hospital facility charges (exclusive of physician and ancillary charges) were also calculated and compared between groups. The median of hospital charge per hour per bed was calculated among total study admissions. The ideal boarding time of these patients was set to be ≤2 h and potential savings was calculated based on the total number of boarding hours beyond the cutoff standard (2 h).

### Statistics

Student’s t Test was used to compare continuous variables between two groups, while Pearson Chi-square (χ2) analysis was used to compare categorical variables. A univariate logistic regression was used initially to determine the Odds Ratio (OR) of each variable potentially affecting prolonged boarding time. A multivariate logistic regression analysis was then used to identify independent risks (Adjusted Odds Ratios, AOR) while avoiding potential confounders. Correlation co-efficiency (r) analysis was conducted between the boarding time and the hospital charge. A scatter-gram with the regression line was developed. |r| ≥ 0.5 is considered a strong relationship. All descriptive and statistical analyses were performed using Stata 12.0 (College Station, TX). A *p* value less than 0.05 was considered statistically significant.

## Results

**Fig. 1:**
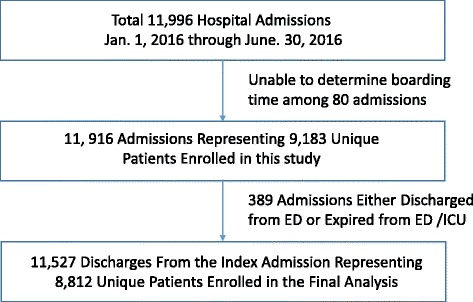
Study Flow Diagram

A total of 11,996 admissions was noted during the period Jan 1, 2016 through June 30, 2016. Of the total 469 either had indeterminate discharge boarding times, experienced discharge directly from ED, or expired while in ED / ICU. Therefore, 11,527 admissions having final dispositions were entered into the analysis (Fig. 1). Median boarding time was 2.1 h [IQR(Interquartile Range) 1.25–3.56 h]. Forty-eight percent of admissions (5494/11,527) had boarding times within 2 h. When hospital admissions were divided into two (regular versus prolonged boarding time) groups, it seemed that relatively older patients, patients with more severe disease(s), patients eventually transferred to other facilities (e.g., skilled nursing facility), or patients discharged to home requiring home health services were predominant in the prolonged discharge boarding time group (Table 1).

**Table 1:** General Information of Study Population

	Regular Boarding (2 h)	Prolonged Boarding (> 2 h)	*p*
	*N* = 5494	*N* = 6033	
Patient general demographics			
Age (years) --- mean (SD)**	49 (15)	52 (16)	< 0.001
Gender (male) --- yes % (n)*	57 (3119)	55 (3289)	0.02
Race**			
African American	30 (1639)	28 (1677)	< 0.001
Caucasian	42 (2304)	47 (2814)	
Others^a^	28 (1551)	26 (1542)	
Ethnicity*			
Hispanic	27 (1489)	25 (1495)	0.01
Not-Hispanic	73 (4001)	75 (4531)	
Others^b^	0.1 (4)	0.1 (7)	
Clinical / operational variables			
PCP assigned (yes)--- % (n)*	49 (2676)	47 (2812)	0.02
APR-DRG SOI --- mean (SD)**	2.5 (0.8)	2.7 (0.8)	< 0.001
median (IQR)**	3 (2–3)	3 (2–3)	< 0.001
Boarding time (h) --- mean (SD)**	1.2 (0.5)	4.7 (6.5)	< 0.001
median (IQR)**	1.2 (0.8–1.6)	3.5 (2.6–5.0)	< 0.001
Interval between case manager consult and disposition (h) --- mean (SD)	4.2 (16)	5.3 (9)	< 0.01
Disposition**			
Home	89 (4878)	72 (4314)	
Skilled nursing facility	4.4 (240)	12.8 (769)	
Home with home health service	2.9 (158)	4.6 (280)	
Others^c^	0.3 (14)	0.2 (9)	
Expired	0.1 (3)	0.1 (97)	< 0.001
Transfers^d^	1.1 (60)	6.3 (380)	
Hospice	0.9 (49)	1.7 (102)	
Court / law enforcement	1.7 (92)	2.8 (170)	
Number of medications prescribed upon disposition (n) --- mean (SD)**	5 (4)	6 (5)	< 0.001
Homeless (yes) --- % (n)**	7.7 (424)	5.7 (344)	< 0.001

Abbreviation: *N* number, *SD* standard deviation, *PCP* primary care physician, *APR-DRG* all patient refined diagnosis related groups, *SOI* severity of illness, *IQR* interquartile range, *h* hour

^a^Native Hawaiian, Asian, American Indian, Patient refused, and Unknown;

^b^Unknown and patient refused;

^c^left against medical advice, organ donation

^d^psychiatric hospital, veterans affairs hospital, designated cancer centers, or other hospitals

*p < 0.05; **p < 0.01

Potential independent risks affecting prolonged discharge boarding were analyzed separately and then added together for multivariate logistic regression analysis. Independent risks affecting prolonged discharge boarding included severity of disease(s) (APR-DRG Severity of Illness), type and time of disposition, and timeliness of case manager / social worker consultation if required (Table 2). Type of patient dispositions played an important role accounting for the top three most significant independent risks for prolonged discharge boarding: (1) transfer to psychiatric hospital, Veterans Affairs hospital, designated cancer centers, or other hospitals with AOR of 7.45 (95% CI 5.35–10.37); (2) transfer to court or law enforcement custody with AOR of 2.51 (95% CI 1.84–3.42); (3) and transfer to skilled nursing facility with AOR of 2.48 (95% CI 2.10–2.93). AOR was 0.57 (95% CI 0.47–0.71) when disposition order was placed during office hours (0800–1700). Analysis of case manager/ social worker consultation for special needs found that prolonged discharge boarding was affected more so in patients receiving late consults (AOR 1.73, 95% CI 1.03–2.89) versus early consults (AOR 1.52, 95% CI 1.37–1.68).

**Table 2:** Odds Ratios of Different Variables Predictive of Prolonged Boarding Time

	Unadjusted Odds Ratio (95% CI)	Adjusted Odds Ratio (95% CI)
Severity of Illness		
Minor (reference)		
Moderate	1.21 (1.03–1.41)	1.11 (0.95–1.31)
Major	1.49 (1.27–1.73)	1.26 (1.07–1.48) *
Extreme	2.78 (2.30–3.36)	1.94 (1.59–2.37) *
Disposition		
Home (reference)		
Skilled nursing facility	3.62 (3.12–4.21)	2.48 (2.10–2.93) *
Home with home health service	2.00 (1.64–2.45)	1.81 (1.46–2.23) *
Others	0.73 (0.31–1.68)	0.67 (0.25–1.81)
Expired	2.64 (0.68–10.21)	1.43 (0.36–5.63)
Transfers	7.16 (5.44–9.43)	7.45 (5.35–10.37) *
Hospice	2.35 (1.67–3.32)	1.48 (1.02–2.15) *
Court / law enforcement	2.09 (1.62–2.70)	2.51 (1.84–3.42) *
Case manager consult		
No consult (reference)		
Early consult	1.75 (1.61–1.89)	1.52 (1.37–1.68) *
Late consult	3.56 (2.18–581)	1.73 (1.03–2.89) *
Homeless	0.72 (0.62–0.84)	0.58 (0.48–0.69) *
Patient disposition order placed between 0800 and 1700	0.66 (0.56–0.78)	0.57 (0.47–0.71) *
Primary care physician assignment	0.92 (0.85–0.99)	
Number of medications at disposition	1.04 (1.03–1.05)	

Hosmer-Lemeshow goodness of fit test: χ^2^ (10) = 9.82, *p* = 0.20. Abbreviations: *CI* confidence interval

*Adjusted odds ratios demonstrated statistical and clinical significance (*p* < 0.05)

A total of 1031 discharged patient after-care satisfaction surveys were received from National Research Corporation (NRC). We specifically reviewed patient responses regarding the quality of discharge instructions and follow-up plans given by hospital providers. Eighty-eight percent (433/494) of patients discharged within the regular (≤ 2 h) period provided positive (i.e., “yes”) responses as compared with 86% (462/537) of those within the prolonged (> 2 h) period (*p* = 0.44). This indicates that regular (efficient) discharge time did not prevent hospital providers from delivering quality discharge instruction and follow-up explanations to patients.

**Fig. 2:**
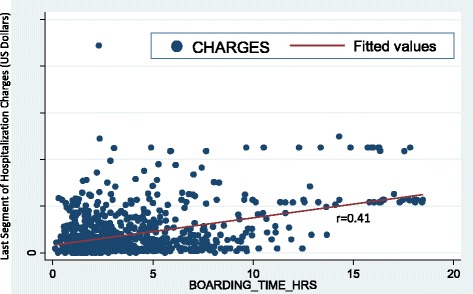
Association Between Hospital Charge and Discharge Boarding

Hospital cost was also calculated during the last segment of patient hospitalization (i.e., any cost charged between discharge disposition order time and end of inpatient bed occupancy interval). In order to minimize confounders, boarding time was right truncated at 18 h to delete outliers. Ninety-nine percent (11,408/11527) of admissions were included in the analysis. A scatter-gram was developed to determine the association between the last segment of hospital charge and the amount of boarding time with its regression line. No strong association was found (Fig. 2) as demonstrated by the weak correlation co-efficiency (*r* = 0.41). A median cost of US $6.00 (IQR 2–18) per hour per bed was charged from this segment of hospitalization resulting in a total 6-month extra-cost of US $ 98,256.00. However, if boarding time can be limited to 2 h per admission, a total of 16,376 hospital bed hours (682 bed days) can be saved based on this study.

## Discussion

In this study, we found that average boarding time (i.e., discharge disposition order time to end of inpatient bed occupancy interval) was a little over 2 h. When considering 2 h as a standard threshold under which inpatient discharge boarding time is reasonable, nearly half of study patient encounters reached the goal. Patient severity of illness, type and time of disposition, and case management timely consultation seemed to affect discharge boarding. Specific attention should be paid to those who require transfer to skilled nursing facilities, psychiatric facilities, Veterans Affairs hospitals, court/law enforcement custody, and/or home health or hospice services. Facilitating case management consultation as early as possible (e.g., ≥ 24 h prior to expected disposition time) will minimize prolonged boarding. Though we are uncertain on the direct link between patient satisfaction and prolonged discharge boarding, efficient discharge (≤ 2 h) does not appear to negatively affect the ability of providers to deliver quality discharge instructions and follow-up plans explanations. In this study, prolonged discharge boarding had a weak association with increased hospital charges. Establishing an inpatient discharge boarding time threshold of less than 2 h would result in saving over 16,000 hospital bed hours (or 680 bed days) at the study hospital. Our findings identified potential risks related to hospital discharge boarding and estimated potential savings when the ideal discharge boarding time is reached. This study adds additional evidence to the current literature regarding further emphasis relative to the necessity of implementing interventions that minimize delays in the inpatient disposition to discharge phase of care.

Delayed hospital discharges typically prolong hospitalization (i.e., increased length of stay), are often multifactorial, and hard to control [16, 17, 21, 22]. Countering delayed discharge boarding requires adequate preparation that begins well before the final disposition. This can usually be predicted and is therefore often easy to control. Hospital operations outcomes can be positively impacted through significant reduction in average bed hours per inpatient encounter thereby maintaining unobstructed admitted patient outflow from the ED. Results of this study also indicate patient satisfaction specifically relative to discharge planning is not negatively impacted by an efficient inpatient discharge process. To the best of our knowledge previous studies focused their investigations on delayed hospital discharges as opposed to a focus on prolonged discharge boarding. Our study is by far one of the larger sample size studies intended to examine a variety of risk factors. It demonstrates robust predictability regarding contributors to prolonged hospital discharge boarding and is quite different from those traditional delayed hospital discharge projects reported.

Different dispositions including transfer of patients to other facilities affect prolonged boarding significantly. These findings were consistent with previous delayed discharge studies [17, 18, 21, 23]. Our findings serve as external validation of prior studies with respect to specific subpopulations (e.g., geriatric, psychiatric) as well as an extension to the general patient population. A significant number of study patients required case manager / social worker consultations and late consultations during their hospitalizations were also associated with prolonged discharge boarding. We recognize the real value that case managers / social workers bring to many patients and families by establishing special follow up resources for patients with high psychosocial risks and/or those requiring short and long term collaborative care after initial recovery from acute illness [24–26]. Due to limited information, we are unable to fully appreciate each of the specific indications for the consultations in this study population. Although reasonable to assume that correlation might occur between the specific types of dispositions and case manager / social worker consultations, our multivariate logistic analysis showed these two risks were relatively independent indicating a variety in scope of consultations. Furthermore, it might appear intuitive to explain the convenience of discharging patients during office hours (0800–1700) since all the other ancillary services are typically in place to assist in facilitating the discharge process.

As for homeless patients, since the study hospital supports special programs targeting this population thereby facilitating their near universal access to healthcare, a reduced discharge boarding bias may be present in our study. Tran et al. reported that placing a pharmacist at bedside to facilitate discharge prescription preparation can potentially decrease discharge time but they did not specifically address the numbers of medications prescribed per patient [22]. Our study showed no significant impact on prolonged discharge boarding as the average number of discharge medications per patient was similar and the majority of medications were e-prescribed and electronically filed to the hospital pharmacist within the study hospital. Further study will delineate the role of these programs / interventions in facilitation of the hospital discharge process in different patient populations.

Hospital cost for discharge boarding was minimal whereas freeing up hospital bed hours (days) was significant when setting a target discharge boarding interval of ≤2 h. Through understanding the relative impact of individual contributors to delayed discharge intervals, meaningful offsetting interventions can be developed, implemented, and tracked to achieve the 2-h threshold thereby arriving at a more resource efficient and fiduciarily responsible future state that better serves our patients. Consistently meeting the 2-h discharge boarding threshold will not negatively impact patient satisfaction based on our review of perception of quality regarding the discharge education process.

Interventions to facilitate improvement in delayed hospital discharge were investigated in many studies [22, 27, 28]. Interventions specifically designed to reduce prolonged discharge boarding times were limited. Our future prospective study will be focused on validating these potential risks and implementing interventions to minimize discharge boarding intervals.

### Limitation

As a retrospective study using hospital admission data from a single urban publicly funded hospital, the methodology may have potential bias in terms of accuracy of information, incomplete data, and potential selection bias due to convenience sampling from one institutional database. Risks that predict prolonged hospital discharge boarding are multi-factorial and contributing factors unknown to us may have influenced our results. Risk of case manager / social worker consultation might not be accurate enough since the reasons for these consults were unclear. Due to the nature of this study design, we are unable to determine the availability of the nursing staff / clerks facilitating hospital discharge process. Confounders may exist among homeless patients with other special programs integrated in the hospital discharge process and should be interpreted with caution. As such, a prospective external multicenter validation study is warranted.

## Conclusions

The average discharge boarding interval in an acute tertiary care hospital was approximately 2 h. Type of dispositions, case management and/or social work timely consultation, and time of day when discharging patients can potentially affect discharge boarding time. An efficient discharge process did not affect patient satisfaction regarding perception of the quality of discharge instructions and follow-up plans explained by providers. Whereas, prolonged discharge boarding has significant negative impact to overall hospital resources and finances.

## Abbreviations

AOR: Adjusted Odds Ratio
APR-DRG: All Patient Refined Diagnosis Related Group
CI: Confidence Interval
ED: Emergency Department
EMR: Electronic Medical Record
HMIS: Homeless Management Information System
HUD: Housing and Urban Development
ICU: Intensive Care Unit
IQR: Interquartile Range
NRC: National Research Corporation
OR: Odds Ratio
SOI: Severity of Illness
